# Mitochondrial Oxidative Phosphorylation in Viral Infections

**DOI:** 10.3390/v15122380

**Published:** 2023-12-04

**Authors:** Neeraja Purandare, Esha Ghosalkar, Lawrence I. Grossman, Siddhesh Aras

**Affiliations:** 1 Center for Molecular Medicine and Genetics, School of Medicine, Wayne State University, Detroit, MI 48201, USA; neeraja.purandare@wayne.edu (N.P.); evg11@case.edu (E.G.); lgrossman@wayne.edu (L.I.G.); 2 Department of Obstetrics and Gynecology, School of Medicine, Wayne State University, Detroit, MI 48201, USA; 3 Department of Oncology, School of Medicine, Wayne State University, Detroit, MI 48201, USA

**Keywords:** oxidative phosphorylation, reactive oxygen species, NADH dehydrogenase, succinate dehydrogensase, cytochrome bc1 complex, cytochrome *c* oxidase, ATP synthase

## Abstract

Mitochondria have been identified as the “powerhouse” of the cell, generating the cellular energy, ATP, for almost seven decades. Research over time has uncovered a multifaceted role of the mitochondrion in processes such as cellular stress signaling, generating precursor molecules, immune response, and apoptosis to name a few. Dysfunctional mitochondria resulting from a departure in homeostasis results in cellular degeneration. Viruses hijack host cell machinery to facilitate their own replication in the absence of a bonafide replication machinery. Replication being an energy intensive process necessitates regulation of the host cell oxidative phosphorylation occurring at the electron transport chain in the mitochondria to generate energy. Mitochondria, therefore, can be an attractive therapeutic target by limiting energy for viral replication. In this review we focus on the physiology of oxidative phosphorylation and on the limited studies highlighting the regulatory effects viruses induce on the electron transport chain.

## 1. Introduction

**Figure 1:**
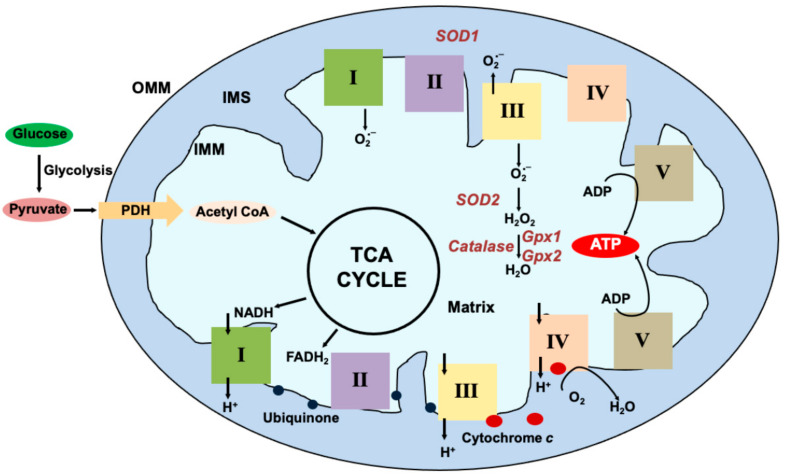
**Mitochondrial Oxidative Phosphorylation:** Glucose, the primary energy substrate is metabolized via glycolysis into pyruvate. From the cytosol, pyruvate enters the mitochondria via the transporters and is decarboxylated by Pyruvate dehydrogenase (PDH) to form Acetyl Coenzyme A (Acetyl CoA) that is used in the Tricarboxylic Acid Cycle (TCA). Reducing equivalents generated in the form of NADH and FADH2 are funneled into Complex I (I) or Complex II (II) embedded in the inner mitochondrial membrane (IMM) respectively. Ubiquinone (blue circles) transfers the electrons from I and II to Complex III (III). Cytochrome c (red circle) transfers the electrons from III to Complex IV (IV) where it is used to reduce molecular oxygen to H_2_O. As the electrons pass through the complexes, protons (H^+^) are pumped into the intermembrane space (IMS) creating a gradient across the IMM. The energy from this gradient is used by complex V (V) also known as ATP synthase to generate ATP from ADP. Some electrons escape and react with molecular oxygen to form superoxide (O_2_^•−^) at I and III. Superoxide is generated on the matrix side at I and both on the matrix and IMS side at III. Superoxide dismutase (SOD) 1 and 2 localized to the IMS and the matrix respectively scavenge the superoxide to generate H_2_O_2_. Glutathione peroxidases further breakdown the peroxide to H_2_O.

Mitochondria have traditionally been viewed as the energy hubs of the cell. The term “powerhouse” was coined almost seven decades ago [1]. Over the last few years this notion has expanded with mitochondria shown to play a moonlighting role in cellular pathophysiology since this organelle is not only vital in cellular metabolism but also in stress response, signaling, immune response, as well as apoptosis. The key ATP generating process in the mitochondria is oxidative phosphorylation (OxPhos), defined as the process wherein energy is generated from nutrients via reduction of oxygen (Figure 1). Viruses, being obligate intracellular pathogens, have to depend on host cells for energy required for replication. OxPhos is therefore one of the main cellular pathways regulated during viral infections. The effector site of OxPhos in the mitochondria is the electron transport chain (ETC), composed of a series of protein complexes embedded in the inner mitochondrial membrane (IMM) containing subunits encoded both on the nuclear and the mitochondrial genomes. Although mitochondria are known to play a vital role in the innate immune response to viral infections, in this review we will focus exclusively on the ETC and how viruses affect the functioning of the OxPhos system.

## 2. Physiology of OxPhos

OxPhos is a key physiological program regulated in cells under viral infections. Four protein complexes make up the ETC, providing the potential energy that drives OxPhos through a fifth complex, ATP synthase. In addition, accessory proteins that interact with these complexes have the capacity to regulate OxPhos. Reducing equivalents (NADH and FADH_2_) generated during glycolysis and the Krebs cycle pass sequentially through the ETC. Coenzyme Q (CoQ) shuttles electrons between Complex I/II and III. Cytochrome *c* transfers the electrons between Complex III and IV. Complex IV, known as Cytochrome *c* oxidase (COX), is the terminal enzyme in the ETC responsible for reduction of molecular oxygen to water using the electrons provided by cytochrome *c*. As the electrons pass through the complexes, a proton gradient is generated across the IMM and, as noted, is used by Complex V (ATP synthase) to generate ATP.

The primary energy substrate utilized by most cells is glucose, which can be metabolized by two crucial pathways. The first of these pathways is glycolysis, which is a series of reactions that occur in the cytoplasm. The main end product of glycolysis is pyruvate, which is shuttled into the mitochondria by the enzyme complex pyruvate dehydrogenase. This complex sits in the inner mitochondria membrane and converts pyruvate to acetyl CoA. The acetyl CoA enters the other major glucose metabolic pathway—the tricarboxylic acid (TCA) cycle, also known as the Krebs cycle. The major role of this cycle is to generate the high energy compounds NADH and FADH_2_ used by the ETC to generate ATP; it also produces metabolic intermediates. The cycle consists of a series of reactions that are catalyzed by a number of dehydrogenase enzymes found in the mitochondria matrix (with the notable exception of succinate dehydrogenase found in the inner mitochondria membrane). The major dehydrogenase enzymes that generate NADH are glyceraldehyde-3-phospate dehydrogenase in glycolysis and isocitrate dehydrogenase, alpha ketoglutarate dehydrogenase, and malate dehydrogenase in the TCA cycle. The NADH and FADH_2_ reducing equivalents generated by the TCA cycle enter the ETC at Complex I and Complex II, respectively. Breakdown of one glucose molecule from the TCA cycle can generate more reducing equivalents as compared to glycolysis: the TCA cycle can generate 6 NADH molecules per glucose molecule whereas glycolysis can only generate 4 NADH. The net energy from glycolysis is reduced further because the first step of glycolysis consumes 2 molecules of ATP. The TCA cycle can provide further reducing equivalents in the form of 2 FADH_2_ molecules and one GTP molecule per glucose molecule.

Other sources that can be utilized by the mitochondria include glutamine and fatty acids. Glutamine, the most abundant amino acid in the blood, enters the cell via transporters on the plasma membrane (SLC38A1, SLC38A2, and SLC1A5). SLC1A5 imports glutamine into the mitochondria, where it is shuttled into the TCA cycle via conversion to alpha ketoglutarate by glutamate dehydrogenase (reviewed in [2]). Fatty acids, on the other hand, are converted into fatty-acyl CoA, which allows them to be broken down in consecutive steps that occur in the mitochondrial matrix to generate several molecules of NADH, FADH_2_, and acetyl CoA depending on the length of the fatty acid. This process is termed beta oxidation. Cardiac myocytes rely primarily on beta oxidation to generate energy. There are other pathways that can provide energy such as the one carbon metabolism (for breakdown of amino acids) and the pentose phosphate pathway that branches off from glycolysis that are beyond the scope of this review. However, all these pathways for breakdown of substrates including glucose, glutamine, or fatty acids and amino acids, ultimately converge upon OxPhos in order to generate ATP required for cellular functioning. Hence, many viruses directly or indirectly target this process to hijack the host cell’s energy metabolism for enhancing their own survival and propagation. Viruses are capable of modulating other steps including substate import, breakdown, and modification for entry into mitochondria (for example, conversion of fatty acids to fatty acyl CoA to enter beta oxidation). However, these are beyond the scope of this review but well summarized elsewhere [3].

One other key function of the ETC is the generation of reactive oxygen species (ROS). Some of the electrons passing through the ETC escape and react with molecular oxygen to form superoxide. Complex I of the ETC generates ROS in the mitochondrial matrix and Complex III generates it in both the matrix and IMS. In an intact physiological system, most of the superoxide generated is reduced by the action of ROS scavengers such as superoxide dismutases and glutathione peroxidases. However, when the ETC is dysfunctional, ROS production exceeds the scavenging capacity, resulting in increased ROS with attendant cellular damage. Total ROS levels, therefore, are the difference between production and scavenging capacity of a cell. Viral infections and ROS have also been discussed here.

## 3. Complex I of the ETC (NADH Dehydrogenase)

Complex I (CI) is made up of 45 subunits with 38 subunits encoded on the nuclear genome and seven on the mitochondrial genome [4]. This complex is identified by its L-shaped structure, with one arm embedded in the IMM and the other protruding into the mitochondrial matrix. Of the 45 subunits, 14 form the core structure (equivalent to the entire complex I in many bacteria) and are equally split between the two arms of the complex. The remaining 31 proteins are considered accessory subunits. While transferring the electrons from NADH to ubiquinone, CI can pump 4 protons across the inner mitochondrial membrane. Enhanced activity of this complex will result in enhanced mitochondrial respiration whereas inhibition would result in excessive ROS production.

## 4. Complex II (Succinate Dehydrogenase, SDH)

This enzyme is a part of both the TCA cycle and the ETC. The SDH complex is made up of four nuclear encoded subunits and is the only one that has no representation on the mitochondrial genome. Subunit A and B are the catalytic subunits, whereas C and D are the membrane anchors. This complex is responsible for the oxidation reaction converting succinate to fumarate. The electrons generated are fed into the ETC. Alternatively, they can be used to reduce the ubiquinone pool and contributes towards antioxidant function [5].

## 5. Complex III (Cytochrome bc1 Complex, CIII)

This complex is made up of 11 subunits with 10 encoded in the nucleus and one in the mitochondria [6]. CIII oxidizes ubiquinol with electrons transferred to cytochrome *c*. Mitochondrial complex III generates superoxide during the ubiquinone Q-cycle [7,8]. During this process, two electrons from CI and CII are transferred to ubiquinone, resulting in its reduction to ubiquinol (QH2). CIII then moves these two electrons to the single electron carrier cytochrome *c*. This results in the unstable radical ubisemiquinone (Q^•−^), which can donate its unpaired electron to oxygen to generate superoxide within the Q-cycle. Also, 2 protons are pumped across the inner mitochondrial membrane to contribute to the electrochemical gradient. In addition to electron transfer, CIII also helps reoxidation of CoQ, and also generates ROS [9].

## 6. Complex IV (Cytochrome *c* Oxidase, COX)

COX is the terminal enzyme in the ETC and is made up of 13 stoichiometric subunits with 10 encoded in the nucleus and three in the mitochondria. More than 90% of the oxygen consumed is reduced to water by COX. Being the rate-limiting enzyme makes COX a vital regulator of the OxPhos system [10]. This complex is unique in that the regulation can occur via multiple complex mechanisms such as allosteric regulation [11], organ specific isoforms [12], and post-translational modifications [13]. This enzyme also plays a vital role in cellular inflammatory pathways [14]. Specific knockdown of subunit 4 isoform 1 (COX4I1) in macrophages has been shown to induce ROS as well as activate pro-inflammatory cytokines [15].

## 7. Complex V (ATP Synthase, CV)

Complex V (ATP Synthase) transforms energy from the proton gradient created by the flow of electrons through the ETC to generate ATP. The nuclear mitochondrial distribution of the subunits that make up this complex is 14:2. The activity of this complex is driven by the proton gradient across the inner mitochondrial membrane to generate energy. The enzyme has two functional domains—one named F_1_, a soluble portion situated in the mitochondrial matrix, and the other F_o_, in the inner mitochondrial membrane. There are 11 genes that form these two domains of which two are encoded by the mitochondrial genome. From these 11 genes, the F1 subunit is comprised of 5 genes and the remaining ones form the F_o_ subunit. An average of 30.63 ATP molecules are formed per glucose molecule via oxidative phosphorylation; by contrast, only 1.45 ATP/glucose molecule is formed by substrate level phosphorylation during glycolysis. An important quantity regarding CV function is its efficiency, the P/O ratio, which is defined as the molecules of ATP generated per molecule of oxygen consumed; the maximum P/O ration for 1 molecule of glucose is 2.79 [16].

## 8. Supercomplexes

Formation of higher order structures, called supercomplexes (SCs), are composed of complex I, III and IV of the ETC and have been identified from yeast to man [17,18]. They are thought to enhance efficiency of OxPhos [19] although contrary evidence has been presented [20]. SCs have defined stoichiometries, for example CI forms a supercomplex with CIII2 and CIV (SC I + III2 + IV, known as the respirasome), as well as with CIII2 alone (SC I + III2). CIII2 forms a supercomplex with CIV (SC III2 + IV), and CV forms dimers (CV2) [21]. Almost all of Complex I is exclusively detected as a part of various SC assemblies [22] whereas complex III can be found as homodimer and complex IV either as a homodimer or monomer. Besides the respirasome, other assemblies include CI + CIII2 and CIII2 + CIV. CI, which is present as a part of CI + CIII2, is much lower than the respirasome [22]. These configurations are important in lower organisms such as yeast, which lack a traditional complex I enzyme. Various subunits from each complex interact with each other to stabilize the supercomplexes. For example- in a CI + CIII2 assembly, there are 2 main interactions—one in the NDUFA11 and the UQCRB, UQCRQ, and UQCRH subunits of CIII, and a second one in the matrix between NDUFB4, NDUFB9, and the CIII subunit UQCRC [23]. On the other hand, the contacts formed between CI and CIII within the respirasome involve so-called supernumerary subunits. These supernumerary subunits are not found in bacteria and are considered to be eukaryotic origin [24].

There are several hypotheses that aim to explain the presence and role of supercomplexes. One of the most prevalent theories is that of they may be useful for substrate channeling. That is, the formation of complexes of enzymes that act sequentially in a pathway so that a specific substrate can be transferred from one enzymatic activity to the next without allowing free diffusion of the substrate into the bulk solution. In order for substrate channeling to occur, a dedicated pool of bound electron carriers (ubiquinone and cytochrome *c*) must be present. However, structural analyses reveled that the distance between the two cytochrome *c* binding sites on CIII and CIV in the supercomplex is too large (>6 nm), thereby precluding the substrate channeling hypothesis [25,26]. Other theories include the efficiency of electron transport rather than strict channeling. In this model, the supercomplex simply provides enhances electrostatic interactions where cytochrome *c* can “roll” between complex III and IV and also mix with the free pool. Other presumed functions include enhanced stability to help the assembly of complexes, in particular for the largest of the ETC complexes—complex I. This is called the cooperative assembly model [27]. The plasticity model [28] suggests that supercomplexes formation helps to adapt to changing metabolic requirements, and that supercomplexes prevent electron escape to reduce ROS [21]. Structurally, some supercomplexes are known to affect membrane curvature and shape. Complex V homodimers have been identified in yeast and appear important for IMM bending and cristae formation [29]. Though it was recently shown to participate in supercomplex formation [30] in a ciliate protist (*Tetrahymena*) and to affect membrane curvature, it is yet to be identified in mammalian supercomplexes.

There are known assembly factors that help to connect these complexes. These include cardiolipin, PHB1 (prohibitin), PHB2, and SCAF1 (supercomplex assembly factor 1) [30]. Of these, SCAF1 (also known as COX7A2L) is the only dedicated assembly factor for supercomplexes and is required for biogenesis and assembly of CIII_2_ + IV but does not affect the assembly of the respirasome [31]. A recent study also showed that, besides SCAF1 containing complexes (S-MRC, SCAF1 containing mitochondrial respiratory chain complex) a second type is also present, called C-MRC (COX7A2 containing mitochondrial respiratory chain complex) is also present. The SCAF1-dependent S-MRC includes the SCAF1-containing respirasome, which accounts for approximately half of total CIII and CIV levels. The remaining CIII and CIV are equally distributed between the CIII2 + CIV supercomplex and free complexes. The C-MRC organization displays a relatively low amount of the COX7A2-containing respirasome, no CIII2 + CIV supercomplex, and abundant free CIII (~60% of total CIII) and CIV (~80% of total CIV). The exclusive presence of one configuration or the other in knockout cells of the corresponding isoform led to some changes in mitochondrial bioenergetics. However, no differences in respiratory parameters were observed where the two MRC organizations co-exist [27]. There are several more details that have been identified regarding super complex components, assembly, and possible functions that are well reviewed elsewhere [32].

## 9. Viruses and Oxidative Phosphorylation

Viruses, being intracellular pathogens, depend on host cellular machinery and energy to facilitate their entry, replication, and exit. In the recent past, significant advances have been made towards understanding the role of cellular mitochondrial function and immune responses [33,34,35]. Studies have also been focused on the crosstalk between mitochondrial dynamics, including fusion-fission and mitophagy (reviewed in [36,37]). Although mitochondrial OxPhos regulates all these functional pathways, very few studies have evaluated the effect and the underlying mechanism of how viruses hijack the host mitochondrial OxPhos system. Here we will review the studies characterizing the effects of viruses on the ETC, specifically the mitochondrial complexes, ATP levels, and ROS. We will also discuss the details regarding the pathways that appear to regulate the ETC complexes in virally infected cells.

For the purpose of this review, we will use the Baltimore classification of viruses wherein the groups are classified on the basis of the viral genome [38]. Most of the work evaluating the role of mitochondrial OxPhos in viral infections has been done on viruses in group IV (+ sense single stranded RNA).

**(+) ssRNA**: This group of viruses harbors a single stranded RNA genome that produces functional mRNAs. An RNA-dependent RNA polymerase transcribes the genome to generate a polyprotein. Viral or host cellular proteases cleave the polyprotein into individual proteins. This group has eight families with either enveloped or non-enveloped capsids.

***Flaviviruses:*** The viruses that have been studied in some detail for their role in regulating OxPhos are Hepatitis C virus (HCV), Zika virus (ZV), and West-Nile virus (WNV).

*Hepatitis C virus*: One of the earliest pieces of evidence of mitochondrial dysfunction in patients with HCV infection was the identification of antimitochondrial antibodies in serum [39]. Similarly, a defect in OxPhos along with increased oxidative stress markers were observed in liver biopsies from patients with chronic HCV infections [40]. In a transgenic mouse model for HCV genotype 1b strain N, defective activity of CI was observed along with an increase in ROS levels. The Core protein of HCV localizes to the mitochondrial outer membrane to cause enhanced Ca^2+^ flux into the mitochondria, resulting in CI dysfunction and increased ROS [41]. Similarly, using cell lines with inducible HCV replicons expressing the entire HCV polyprotein, enhanced calcium toxicity in the mitochondria was shown to cause an inhibition of CI activity and an increase in ROS [42], which were found to be reversible upon amantadine treatment [43]. It was also hypothesized on the basis of a case report that CIII dependent mitochondrial dysfunction underlies the myopathy phenotype in HCV [44]. HCV non-structural protein NS5A also localizes to the mitochondrial fraction and induces ROS via dysregulation of Ca^2+^ signaling [45,46]. Transcriptomic analysis of Huh-7.5 cells transfected with the full-length HCV genome displayed a reduction in expression of CI (*ND1*, *ND3*, *ND4*) and CIV (*MT-CO2*) subunits encoded on the mitochondrial genome [47]. Interactome analysis has identified HCV core, p7, and NS4B proteins to interact with the mitochondrial proteome in host cells [48]. MNRR1 (CHCHD2), a bi-organellar regulator of mitochondrial function that interacts with CIV and is required for its optimal function, was also identified as one of the top candidate host gene required for HCV replication [48]. MNRR1 was first identified as an HCV Non-structural protein 2 transregulated protein [49]. Although HCV inhibits mitochondria, the induction and requirement of MNRR1 could be hypothesized to be related to its anti-apoptotic or transcriptional regulatory function [50,51].

*Zika virus*: Zika virus rose to prominence in the recent past due to its association with microcephaly [52]. The presence of viral nucleic acids in fetal brains and placentas led to the causal association of microcephaly with viral infection [53]. Although there is a lack of evidence suggesting a direct effect of Zika viral proteins regulating the ETC, studies have shown Complexes II, IV, and V to be affected. Zika viral proteins such as NS4A and 4B do localize to the mitochondria to modulate mitochondrial dynamics and apoptosis [54,55].MNRR1 is also upregulated in ZIKV infected cells and may promote viral replication [56]. Zika viral infection of neurons generated the metabolite itaconate from the TCA cycle that inhibits CII activity, resulting in mitochondrial dysfunction [57]. The effect on oxygen consumption rate (OCR), a function of CIV, displayed a strain-specific effect. Using MRC-5 cells, only the MR766 strain was shown to inhibit OCR. Other strains, such as H/PF/2013, M-F37L, DN-1, and DN-2, were comparable to the uninfected cells for their effect on OCR [58]. Finally, Zika viral (and also other flaviviral) capsid proteins induce DAPIT [59], an assembly subunit of CV [60].

*West Nile virus*: This virus infects keratinocytes and dendritic cells in skin as well as cells in the central nervous system [61,62]. Using neuroblastoma cells A172, significant downregulation was observed for nuclear encoded genes for CII (SDHB), CIV (COX5B and 6B), and CV (ATP5G1, 5C1, 5J, 5B, 5A1, 5O, 5F1), suggestive of an inhibitory effect on ETC and mitochondrial function [63]. In virally infected Vero cells, oxidative phosphorylation was inhibited with a shift towards glycolysis [64]. The modulation of other mitochondrial pathways by West Nile virus has been reviewed previously [65,66].

***Coronaviruses:***

*SARS-CoV-2*: The recent COVID pandemic overburdened the economic and health care sectors across the globe. Research was focused towards identifying therapeutic targets and a vaccine. The initial studies performed in multiple cell and tissue types identified an inhibitory effect of viral infection on nuclear encoded CI subunits including *NDUFS2*, *NDUFS6*, *NDUFB7* [67]. CoV-2 was also shown to inhibit both nuclear as well as mitochondrially encoded mitochondrial genes. The gene profile was evaluated across disease progression. At the initial stage, minimal effects on gene expression were observed in lungs. Downregulation of mitochondrial genes was observed when viral titers peaked. The downregulated genes involved those encoding the structural and assembly subunits of the OxPhos complexes. Upon clearing of the virus, the inhibitory effect on mitochondrial genes was reversed in the lung, but not other organs such as the heart, liver, and kidneys [68]. Downregulation of CI was proposed to be responsible for the hypoxemic phenotype associated with the disease [69]. Cytokine storm underlies the pathogenicity of COVID. Monocytes infected with CoV-2 displayed downregulation of subunits from complexes I, II, III, and V, resulting in dysfunctional mitochondria and enhanced ROS that contributed to the cytokine production [70]. OCR was significantly reduced in peripheral blood mononuclear cells from COVID patients [71]. Additionally, multiple viral proteins such as ORF-3C localize to the mitochondria and induce organellar dysfunction [72]. Moreover, NSP10 interacts with ND4L and COXII to modulate complex activity [73]. Enzyme remodeling by subunit switch has also been observed specifically in SARS-CoV-2 infected cells. The C15orf48 subunit is induced upon infection and replaces its paralog, NDUFA4, in CIV [14]. Finally, levels of OxPhos regulators such as MNRR1 were also shown to be lower in SARS-CoV-2 patient hearts and may potentially contribute towards the cardiac complications of the disease [74].

***Others:*** The three other viruses in the + ssRNA group include Rubella virus, Coxsackie B3, and Hepatitis E virus. Rubella virus causing German measles, in contrast to the others in the group, actually induced mitochondrial OxPhos by enhancing activities of CI, II, III, and IV in A549 cells 24 h post infection using isolated mitochondria [75]. Subunits SDHA, SDHB (CI), UQCRC2 (CIII), and COX4I1 (CIV) were also induced upon acute infection. The induction of OxPhos was found to be strain specific with Wb-12 strain showing maximal induction and 07-00426 showing minimal increase [76]. The induction of OxPhos in rubella virus infected cells has been attributed to the energy requirement of viral replication owing to the observation that the mitochondria in the infected cells are in close proximity to the viral replication complex [77]. Host cellular p32 protein facilitates the interaction of viral capsid with the mitochondria [78].

Coxsackie virus B3 (CVB3) mediated effects on OxPhos depend on the immune responsiveness of the host. Studies using C57/BL6 mice (that efficiently eliminates the virus) and A.SW/SnJ (unable to eliminate the virus) show a completely variable response. Hearts from C57/BL6 show an increase in CI and CIII activities whereas A.SW/SnJ hearts show a significant reduction [79] suggesting that mitochondrial function has a potential role to play in the viral replication cycle as well as in the host cellular response to infection.

Hepatitis E virus (HEV) is the causal agent of acute viral hepatitis. Recently, cell culture models have identified CIII function to be required for the replication of HEV [80] making it an attractive drug target. OxPhos dysfunction was also evident in primary human brain microvascular endothelial cells wherein the infected cells displayed a significant reduction in the protein levels of ATP5A1, a catalytic subunit of CV, resulting in bioenergetic deficit and apoptosis [81].

**(-) ssRNA**: The three viruses in this group on which studies have been performed characterizing OxPhos are Influenza, Rabies, and Respiratory syncytial virus (RSV).

*Influenza*: This virus is responsible for causing seasonal epidemics as well as pandemics (reviewed in [82]. One of the early studies documenting the effect of influenzas virus on mitochondrial function identified an ~50% reduction in MDCK cellular oxygen consumption rate in the infected cells compared to the mock control [83]. In contrast, mass spectrometric analysis of A549 cells infected with swine influenza virus identified NDUFS8 and ATP5B and 5D subunits to be upregulated [84] whereas H1N1 infection did not affect protein levels of ETC subunits [84]. Recently, H5N1 influenza viral infected cells were shown to have significantly higher levels of COX subunit 4 isoform 1 (COX4I1). Further, a CRISPR/Cas9 knockout of COX4I1 resulted in a ~200-fold reduction in viral titers. Lycorine, a compound inhibiting viral replication, was shown to function by inhibition of this isoform of COX [85]. Influenza virus may also indirectly affect the expression of certain subunits such as COX6C via regulation of microRNAs [86]. The M1 protein from influenza virus interacts with and inhibits the functioning of CIV [87]. These effects on mitochondrial function suggest that the virus probably hijacks mitochondrial metabolism depending on the stage of its replication cycle such that activation is induced via multiple pathways when energy is required [88].

*Rabies*: This virus, responsible for causing fatal encephalitis, induces mitochondrial dysfunction underlying the pathogenic phenotype. Mitochondrial function was evaluated in baby hamster kidney cells using the challenge virus standard-11 strain. A significant reduction in intracellular ATP levels was observed in these cells along with increased ROS levels. Both of these were attributed to high mitochondrial membrane potential resulting from increased activities of CI and CIV generating ROS and hydrolysis of ATP [89]. The same group later identified rabies viral phosphoprotein to interact with CI and regulate its function [90]. Extensive analysis was also performed on postmortem brain tissues from rabies encephalitis. Increased activities of CI, IV, and V were observed along with an increase in multiple subunit proteins that constitute individual complexes of the ETC [91].

*Respiratory syncytial virus:* This virus causes acute lower respiratory tract infections especially in the young and immunocompromised. RSV infected cells display a perinuclear clustering of the mitochondria suggestive of cellular stress. A time dependent reduction in basal oxygen consumption was observed in A549 cells with an increase in glycolysis and ROS levels [92]. These changes were shown to be CI dependent. Reduced activity of CI along with increased ROS levels were conducive for RSV replication in these cells and these effects were induced by the matrix protein of the virus [93]. A downregulation of mitochondrial biogenesis was also a feature of RSV infected cells [94].

**ssRNA-RT**: This group includes retroviruses with the most common being Human Immunodeficiency Virus (HIV). The viruses in this category have a reverse transcriptase enzyme that generates a cDNA intermediate from the RNA genome. One of the earliest pieces of evidence of HIV virus affecting mitochondrial function was described almost four decades ago. HIV positive ACH-2 cells were shown to have mitochondrially localized viral RNA and proportionally defective mitochondrial morphology [95]. Shortly thereafter, using *Saccharomyces cerevisiae* as a model system, it was shown that the HIV protein Vpr induced mitochondrial dysfunction by reducing activities of the entire ETC [96]. In strong contrast, an increase in expression of individual subunits and activity of CIV was observed [97]. HIV-1 infection also inhibits CI activity by a specific downregulation of the NDUFA6 subunit [98]. PBMCs from non-treated HIV-infected patients were found to have reduced CII, III, and IV activities [99]. We have recently shown in glial cells that the inhibitory effect of antiretrovirals on SDH is abrogated in the presence of latent or active HIV infection [100]. Effects of viral proteins on the ETC as a result of direct interaction have also been described. A direct interaction between the p2 peptide of the Gag and Gag-Pol precursors of HIV and COXI during acute phase of infection results in increased ATP levels [101]. Tat protein of HIV, however, inhibits COX and induces mitochondrial membrane permeabilization [102]. This property has allowed the use of Tat as a COX inhibitor in experimental settings. The ATP synthase β-subunit is required for optimal HIV viral transfer from the antigen presenting cell to the CD4+ T-cells. Although the mechanism of the localization of an inner mitochondrial protein to the cell surface is unclear, these findings made ATP synthase an attractive therapeutic target for HIV [103]. Defects in mitochondrial function (CIV) measured as OCR also depends on the stage of infection. Viral infection proportionally inhibited OCR rates with minimal effects on glycolysis [104]. These contrasting results on ETC in HIV infected cells could potentially point towards cell and strain specific effects. Comprehensive studies towards this avenue are required for a better understanding of how HIV subverts mitochondrial OxPhos towards its replicative benefit. HIV viral proteins also regulate multiple physiological processes of the mitochondria (reviewed in [105]).

**dsDNA-RT**: These viruses have a DNA genome with an RNA intermediate. Hepatitis B (HBV) is an important virus in this group, responsible for liver disease that can lead to cirrhosis and hepatocellular carcinoma. A protein encoded by the HBV genome, ORF X (HBx), interacts with the OMM and induces apoptosis [106,107]. Using a two-hybrid assay system it was also shown that HBx interacted with subunit 3 of CIV (COXIII) [108]. This results in an increase in mitochondrial function and cell growth [109]. A significant downregulation of the ETC complex levels along with activity was observed in hepatoma cells expressing HBx [110] with a resultant increase in ROS levels. A ~50% reduction in CII activity was also associated with chronic HBV infection as evaluated using liver biopsy specimens [111]. HBV, in contrast, induces OxPhos in macrophages and this increase is required to downregulate the immune response [112]. HBV DNA also can integrate into the mitochondrial genome coding for the subunits of ETC and may contribute towards organellar dysfunction in infected cells [113]. Correcting mitochondrial dysfunction is a potential therapeutic target in chronic HBV [114]. These results indicate that the virus differentially regulates mitochondrial function in cell types conducive towards its own replication. In some cells it increases mitochondrial function, whereas in others it decreases them with enhanced ROS.

**dsDNA:** Three DNA viruses have been studied for their effects on mitochondrial OxPhos and are described here.

*Human Cytomegalovirus (HCMV)*: This herpesvirus is highly seroprevalent in the population. A majority of HCMV infections are congenital and result in neurodevelopmental anomalies [115]. HCMV depends on host cell energy for its replication. HCMV infected cells induce both OxPhos and glycolysis [116]. Metabolomic analysis also show an increase in the TCA cycle as well as glycolytic intermediates, supporting the induction of OxPhos and glycolysis [117]. A viral protein, pUL13, is responsible for the effect on OxPhos since virus with a deletion of pUL13 fails to induce OxPhos. pUL13 has been shown to interact with the MICOS complex responsible for maintenance of cristae that harbor the individual OxPhos complexes [118]. Another viral protein, pUL37x1, induces mitochondrial biogenesis and contributes towards OxPhos induction [119]. Viral infection also induces factors critical towards maintenance of the mitochondrial genome as well as those responsible for the assembly of the individual OxPhos complexes and for mitoribosome biogenesis [120]. Finally, GRIM-19 (Gene associated with retinoic acid and interferon-β-induced mortality-19) is another assembly factor of CI [121]. This protein relocalizes to other cellular niches such as the nucleus in response to mitochondrial stress to induce apoptosis. In HCMV infected cells, the β2.7 RNA transcript was shown to interact with GRIM19 to prevent its nuclear localization and thereby inhibit apoptosis of the infected cells [122].

*Epstein-Barr virus (EBV)*: EBV is also seroprevalent with latent infection. Conditions of immunosuppression result in infection [123]. During early stages of infection, an induction of glycolysis takes place [124]. As the infection proceeds, OxPhos induction also occurs indirectly via activation of one-carbon metabolism [125]. One carbon metabolism is a series of reactions providing methyl groups for a multitude of cellular pathways including OxPhos [126]. Additionally, like CI, CII also has SDUFA1-4 that are responsible for the assembly of the complex [127,128,129]. However, recent studies have identified SENP2 to regulate sumoylation and assembly of CII under nutrient stressed condition [130]. This study identified desumoylation of SDHA subunit of CII under conditions of glutamine deprivation to result in an inhibitory effect on CII assembly and function. Epstein-Barr Viral (EBV) protein LMP1 reduces functioning of SENP2 [131]. However, this study did not evaluate the effect on mitochondrial function.

*Human Papilloma virus (HPV)*: HPV, the causal agent of cervical cancers, also regulates host cellular OxPhos. The E2 protein plays a key role in viral genome replication [132]. E2 from high-risk HPV-16 and 18 interacts with UQCRC2 and UQCRFS1 (CIII) and COXII (CIV) to induce ROS generation by the mitochondria [133]. Recently, cells stably expressing the oncoprotein E7 of HPV-16 was shown to interact with the ATP5B subunit of CV, causing an increase in mitochondrial function. A mild increase was also observed with E7 of HPV-8 [134]. The E2 protein also regulates mitochondrial function indirectly via induction of p32 [135], an RNA-binding protein associated with TFAM [136]. TFAM is required for mitochondrial transcription and translation (reviewed in [137]).

## 10. Viruses and Mitochondrial Reactive Oxygen Species

Multiple studies have reported the generation of ROS upon direct or indirect (for example via gene regulation) interaction of viral proteins with the host cell mitochondria. Examples are HCV mediated inhibition of CI activity [42], downregulation of assembly factors for CIII in SARS-CoV-2 infected cells [138], and Rabies viral phosphoprotein interaction with CI induce ROS production [90]. Others have reported an increase in mitochondrial ROS via (a) regulation of proteins involved in cristae structure such as prohibitins [139], (b) dysregulated calcium homeostasis resulting in a mitochondrial overload and ROS generation as seen with HBsAg, the surface antigen of HBV [140], (c) regulation of the mitochondrial membrane channels resulting in membrane depolarization and ROS by Tat protein of HIV [102], and (d) downregulation of the ROS scavenging enzymes such as SOD2 as seen in SARS-CoV-2 infections [138].

Excessive ROS is deleterious to the host cell and therefore would not be conducive for viral replication. Therefore, the ROS generated must be within levels that can facilitate viral replication and prevent host cell death. So why do viruses induce ROS unless it’s beneficial? The role of ROS as a signaling molecule [141] in the host cells could underlie the induction observed in virally infected cells.

The two major reactive species generated by the mitochondria are the superoxide anion (O_2_^•−^)and hydrogen peroxide (H_2_O_2_) [142]. Superoxide anion, for example, has been shown to activate the Raf/MEK/ERK pathway [143]. This pathway is required for replication of SARS-CoV-2 [144]. H_2_O_2_ activates the p38-MAPK pathway to facilitate replication of HCMV [145]. HCV induced ROS also facilitates viral replication via NFκB-dependent induction of DR6, which interacts with the viral protein NS5A to induce viral replication [146]. Similarly, studies have shown increased ROS to stabilize HIF-1 [147,148]. HIF-1 causes enhanced infectivity and replication of HIV in host cells [149,150].

ROS, in addition to regulating cellular signaling pathways, can also modify viral proteins to enhance its functionality. Oxidation induces dimerization and guanylation of the NS5A protein of Dengue virus, enhancing RNA-capping and replication [151]. Methionine oxidation of Kaposi Sarcoma Herpes Viral helicase also enhances its stability and function [152]. Although ROS are beneficial for some viral infections, high levels of ROS would be deleterious to the host cell and therefore result in abortive replication of the virus. Thus, viruses also induce antioxidant genes when the ROS levels in cells reach levels to activate apoptotic cascades. HPV E7 protein induces the enzyme catalase to degrade H_2_O_2_ [153]. Similarly, HBV induces NRF2 to activate antioxidant genes [154]. NS5A of HCV induces Glutathione peroxidase 1 (GPX1) and GPX4. Induction of GPX4 counteracts lipid peroxidation, resulting in enhanced infectivity of the progeny virus [155]. Viruses such as HCMV [156,157] and Influenza (reviewed in [158]) induce ROS acutely to facilitate induction of viral promoters and then induces ROS scavengers to reduce ROS. As ROS also induces apoptosis, viruses counteract the apoptotic pathway by multiple mechanisms such as transcriptional inhibition of proapoptotic proteins like Bim by EBNA3A and EBNA3C of EBV [159], or induction of proteins that inhibit multiple targets in the apoptotic cascade (reviewed in [160]).

## 11. Summary

In summary, although an exact mechanism is lacking, there appears to be a fine regulatory system in play to ensure optimal viral replication and evasion of immune response in the host cell. Viruses either induce or inhibit OxPhos, depending on its life cycle, either by direct interaction with the OxPhos complexes and their assembly factors or indirectly by regulating transcription of specific subunits and assembly factors. As ROS is a product of ETC function, viruses also regulate ROS generated via the ETC to support their own replication and modulate host signaling pathways. Table 1 summarizes the effects observed on OxPhos in virally infected cells. Finally, detailed studies characterizing a common mechanism used by multiple viruses are required. Mechanistic studies on mitochondrial supercomplexes would help uncover novel molecular mechanisms hijacked by viruses. This would allow the characterization of potential therapeutic targets for viral infection that would be of immense benefit during viral pandemics.

## Figures and Tables

**Table 1:** Effects of different viruses on OxPhos complexes.

Group	Virus	Complex	Effect	Reference
**(+) ssRNA**	CVB3	I	Induces	[161]
	CVB3	III	Inhibits	[161]
	HCV	I	Inhibits	[42]
	HCV	III	Inhibits	[44]
	HEV	III	Induces	[80]
	Rubella Virus	II, III	Induces	[75]
	Rubella Virus	IV	Inhibits	[75]
	SARS-CoV-2	I	Inhibits	[69]
	SARS-CoV-2	III	Inhibits	[70]
	ZIKV	II	Inhibits	[57]
	ZIKV	V	Induces	[59]
	West Nile Virus	II	Inhibits	[63]
**(-) ssRNA**	H5N1 Virus	IV	Induces	[69]
	Influenza Virus	II	Induces	[162]
	Influenza Virus	III	Induces	[163]
	Influenza Virus	V	Induces	[164]
	Rabies Virus	I	Induces	[90]
	Rabies Virus	IV	Induces	[89]
	RSV	I	Inhibits	[165]
**ssRNA-RT**	HIV	I	Inhibits	[98]
	HIV	III	Inhibits	[97]
	HIV	IV	Induces	[101]
	HIV	IV	Inhibits	[102]
	HIV	V	Induces	[103]
**dsRNA-RT**	HBV	I, III, IV, V	Inhibits	[110]
	HBV	II	Inhibits	[111]
**dsDNA**	HCMV	I, II, III, IV, V	Induces	[116]
	EBV	II	Inhibits	[131]
